# Perturbations of tryptophan catabolism via the kynurenine pathway are associated with stage 2 postoperative outcomes in single ventricle heart disease

**DOI:** 10.14814/phy2.70133

**Published:** 2024-11-24

**Authors:** Jennifer Romanowicz, Sierra Niemiec, Ludmila Khailova, Tanner Lehmann, Christopher A. Mancuso, Max B. Mitchell, Gareth J. Morgan, Mark Twite, Michael V. DiMaria, Jelena Klawitter, Jesse A. Davidson, Benjamin S. Frank

**Affiliations:** ^1^ Department of Pediatrics, Section of Cardiology Children's Hospital Colorado and University of Colorado Anschutz Aurora Colorado USA; ^2^ Department of Biostatistics and Informatics University of Colorado Anschutz Aurora Colorado USA; ^3^ Department of Cardiac Surgery Children's Hospital Colorado and University of Colorado Anschutz Aurora Colorado USA; ^4^ Department of Anesthesiology University of Colorado Anschutz Aurora Colorado USA; ^5^ Congenital Heart Center University of Michigan C.S. Mott Children's Hospital Ann Arbor Michigan USA

**Keywords:** cardiac surgery, congenital heart disease, Glenn, kynurenic acid, serotonin

## Abstract

Preliminary evidence suggests perturbations of the kynurenine pathway (KP) of tryptophan metabolism in infants with single ventricle heart disease (SVHD). In 72 infants with SVHD undergoing stage 2 palliation (S2P) and 41 controls, we quantified serum KP metabolite concentrations via tandem mass spectroscopy pre‐S2P and post‐S2P at 2, 24, and 48 h and assessed metabolite relationships with post‐S2P outcomes (length of stay, hypoxemia burden, and intubation duration). Pre‐S2P, SVHD infants had lower tryptophan and serotonin levels and higher kynurenic acid, 3‐hydroxykynurenine, and picolinic acid levels than controls. Post‐S2P, metabolites peaked at 2 h, with return to baseline by 48 h for all except kynurenic acid, which remained elevated. Metabolite concentrations pre‐S2P were poorly associated with outcomes. A lower serotonin peak 2 h post‐S2P was associated with longer length of stay and intubation duration. Multiple metabolites at 24 and 48 h correlated with outcomes; notably, elevated kynurenic acid was associated with worse results for all three outcomes. Our results confirm that interstage SVHD infants have altered KP activity compared to controls. Further, the link between outcomes and KP metabolites post‐S2P—but not at baseline—demonstrates that acute, perioperative changes in tryptophan catabolism may be more important to tolerating S2P physiology than chronic interstage changes.

## 1 INTRODUCTION

Single ventricle heart disease (SVHD) encompasses a range of severe congenital heart defects that result in the child having only one functional pumping chamber. There is no method for definitive repair, and children with SVHD must undergo a series of 2–3 palliative surgeries over the first 3–4 years of their lives for a chance at survival. Mortality risk is significant, with a transplant‐free survival rate of only 61% at 6 years of age (Newburger et al., 2018). Morbidity comes in many forms, affecting nearly every organ system in the body, with the brain, kidneys, and gut most notably at risk during early childhood (Blinder et al., 2012; Newburger et al., 2018; Patterson et al., 2017; Schuchardt et al., 2018; Weiss et al., 2011).

Stage 2 palliation (S2P) refers to the construction of a superior cavopulmonary anastomosis, which connects the superior vena cava to the pulmonary arteries, often as the sole source of pulmonary blood flow. This surgery is done during infancy, typically at 4–6 months of age (Meza et al., 2017). The completion of this surgery marks the end of the “interstage” period, which is the period of highest mortality risk for the SVHD population (Newburger et al., 2018). Post‐S2P, SVHD patients have passive pulmonary blood flow (i.e., no sub‐pulmonary ventricle) for the first time. Many patients tolerate this change in physiology, but others experience hypoxemia, development of collateral vessels, and increasing central venous pressure due to inadequacy of the pulmonary vascular bed and the resulting elevated pulmonary vascular resistance. Thus, optimal pulmonary vascular development is crucial both for immediate post‐S2P outcomes, and ultimately, the long‐term success of the single ventricle palliation strategy. Risk‐stratification of infants most vulnerable to poor postsurgical outcomes remains difficult.

**FIGURE 1:**
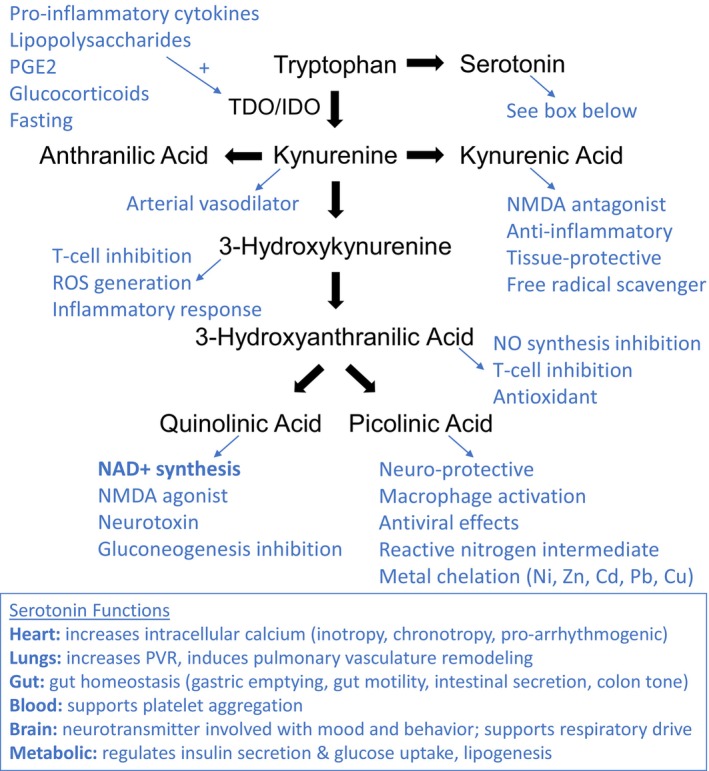
Tryptophan catabolism pathways including the serotonin pathway and the kynurenine pathway (KP). The primary purpose of the KP is believed to be providing precursors for NAD+ synthesis given preferential shunting toward quinolinic acid. KP metabolites have numerous biological activities, especially pertaining to immune regulation, inflammation, and neuromodulation. Cd, cadmium; Cu, copper; IDO, indoleamine‐2,3‐dioxygenase; NAD+, nicotinamide adenine dinucleotide; Ni, nickel; NMDA, N‐methyl‐D‐aspartate; NO, nitric oxide; Pb, lead; PGE2, prostaglandin E2; TDO, tryptophan‐2,3‐dioxygenase; XA, xanthurenic acid; Zn, zinc.

Metabolomic analysis has emerged as a useful tool in the assessment of the perioperative response to cardiopulmonary bypass (CPB) and cardiac surgery, offering a way to examine the end result of both gene and protein expression at a single point in time. Our group has previously demonstrated a distinct global metabolomic fingerprint for infants with SVHD, compared to biventricular controls (Frank et al., 2023). Further, we have identified that elevated kynurenic acid—a byproduct of the kynurenine pathway (KP) of tryptophan metabolism—is associated with death, prolonged hospital stay, and prolonged intubation in a pooled population of infants undergoing CPB for any cardiac diagnosis (Davidson et al., 2018). Tryptophan catabolism produces numerous biologically active metabolites via two main pathways: the KP (>95%) and the serotonin pathway (Cervenka et al., 2017) (Figure 1). The KP is upregulated in proinflammatory states and has numerous immunosuppressive effects (Cervenka et al., 2017; Wirthgen et al., 2018). The end product of the KP is quinolinic acid, which is the primary source of de novo production of nicotinamide adenine dinucleotide (NAD+). Increased KP activity has been observed in numerous disease states; most notable to the SVHD population is pulmonary hypertension. KP intermediates inhibit endogenous nitric oxide synthesis (Oh et al., 2004), and a link between KP activation and early pulmonary hypertension development is established in an adult population as well as in preclinical studies (Simpson, Ambade, et al., 2023; Simpson, Coursen, et al., 2023).

On global metabolomic analysis of SVHD infants during the interstage period, there was evidence supporting dysregulation of selected KP metabolites (Frank et al., 2023). To determine whether the entire KP was active in the perioperative period in SVHD infants, we previously performed a small pilot study utilizing relative quantification of all KP metabolites. This proof of concept investigation revealed early activation of the KP in SVHD infants directly following S2P surgery (Sabapathy et al., 2020). Absolute quantification of KP metabolites in a larger sample size sufficiently powered to evaluate outcomes is necessary to understand associations between KP dysregulation and postoperative physiology after S2P.

The current study aimed to perform complete quantitative mapping of the KP and serotonin pathways in SVHD infants prior to and for the 48 h following S2P operation. We sought to define how baseline (interstage) concentrations differed from those of healthy biventricular controls. Further, we sought to quantify how the concentrations of each metabolite changed in the immediate postoperative period. Finally, we aimed to define how alterations in specific metabolite concentrations related to both preoperative cardiac physiology and postoperative outcomes directly following S2P.

## 2 MATERIALS AND METHODS

This study follows a single‐center prospective cohort design. Permission to conduct the study was granted by the Colorado Multiple Institutional Review Board. Written informed consent was obtained from each subject's legal guardian at enrollment for both cases and controls.

Infants with SVHD were identified at the time of pre‐S2P cardiac catheterization or, if no catheterization was performed, at the time of S2P surgery. SVHD patients were eligible if they were between the ages 31 days and 2 years and planning to undergo S2P surgery. S2P was defined as the construction of any type of superior cavopulmonary anastomosis (e.g., Glenn, hemi‐Fontan) whether or not patients had undergone a stage 1 procedure. SVHD patients were excluded if their S2P plan included maintaining a pulsatile source of pulmonary blood flow (i.e., “1.5 ventricle” repair) or if their weight was less than 4 kg at the time of S2P.

Biventricular controls were identified by reviewing the general surgery schedule for patients undergoing an elective, noncardiac, surgical procedure under general anesthesia that included clinical need for intravenous access. Control patients were eligible if they were between 3 and 12 months old and weighed at least 4 kg at the time of surgery. Potential control patients were excluded if they had known or suspected cardiac, pulmonary, infectious, or genetic abnormalities.

### 2.1 Clinical data

Participant clinical data were extracted from the medical record including demographic information, native anatomy, procedure history, cardiac catheterization data, and post‐S2P outcomes. Catheterization data were obtained prior to S2P and included mean pulmonary arterial pressure (mPAP), Fick‐derived calculation of indexed pulmonary vascular resistance (PVR), and systemic oxygen saturation (SaO_2_) obtained by co‐oximetry. Postoperative hospital length of stay (LOS) was measured in days and intubation duration in hours. Postoperative oxygen saturations—as measured by pulse oximetry—in the cardiac intensive care unit (CICU) were downloaded in 1‐min intervals from the clinical monitoring system (BedMaster, Anandic Medical Systems, Feuerthalen, Switzerland), and hypoxia burden was calculated as the percentage of minutes in the first 48 h that the patient's pulse oximeter read below 70%. All study data were recorded and managed using REDCap electronic data capture tools hosted at University of Colorado.

### 2.2 Kynurenine pathway analysis

For SVHD patients, blood samples were obtained from a systemic vein prior to S2P—either during a pre‐S2P cardiac catheterization or in the operating room prior to first surgical incision if no catheterization sample was available—as well as from a central line in the CICU at 2, 24, and 48 h postoperatively. Timing of samples was aligned to count from the patient's arrival to the CICU. For control patients, venous blood was obtained during peripheral IV insertion prior to the first surgical incision.

Venous blood samples were processed for serum, aliquoted, and stored at −80°C until sent for batch analysis. Kynurenines were analyzed using a modification of the assay described by Zhu et al. (2011). Briefly, 60 μL of plasma samples were enriched with 21.6 μL of an isotope labeled internal standard mix [to a final concentration of 635 nM for d4‐serotonin (Alsachim, Illkirch‐Graffenstaden, France), d5‐kynureninic acid (Sigma‐Aldrich, St. Louis, Missouri, United States), 13C6‐anthralinic acid (Alsachim), 13C3,15N‐3OH kynurenine (Toronto Research Chemicals, Toronto, Canada), and d4‐picolinic acid (Sigma‐Aldrich); and 1950 nM for 13C6‐kynurenine (Alsachim), 13C4,15N‐quinolinic acid (Alsachim) and d5‐tryptophan (Sigma‐Aldrich)]. Then 218.4 μL of methanol were added and samples were vortexed and centrifuged at 20,000 × *g* for 10 min. The supernatant was evaporated to dryness and the residue was reconstituted in 60 μL of water containing 0.1% formic acid. Calibrator standards were prepared in 0.1% formic acid in water as surrogate matrix. Final concentration ranges for calibrators were as follows: tryptophan: 62.5–125,000 ng/mL; kynurenine, kynurenic acid, and anthranilic acid: 1–2000 ng/mL; 3OH kynurenine, picolinic acid, quinolinic acid, 3OH anthranilic acid, and serotonin: 0.25–500 ng/mL. Quality control samples were prepared by enriching blank plasma samples with appropriate volumes of kynurenines to reach low quality control levels of 5× lowest calibrator, mid quality control levels of 37.5% of the highest calibrator and high‐quality control levels of 75% of the highest calibrator. All calibrators and quality control samples contained the same final concentrations of internal standards and were handled in the same way as the patient study samples. LC–MS/MS was performed using an Agilent Technologies (Santa Clara, CA) 1100 HPLC system connected to a SCIEX (Concord, ON, Canada) 6500+ QTRAP mass spectrometer equipped with a turbo ion spray source operated in electrospray mode. 10 μL (Wirthgen et al., 2018) of sample was injected onto an Atlantis T3 3 μm (2.1 × 50 mm) column (with attached 2.1 × 5 mm guard column, both from Waters, Milford, MA). Mobile phase consisted of 0.1% formic acid in water (Solvent A) and acetonitrile (Solvent B). The following 8‐min‐long gradient was run: from 0 to 0.5 min: 98% Solvent A, 0.5 to 4.2 min: 98% to 76% Solvent A, 4.2 to 4.5 min: 76% to 10% Solvent A, from 4.5 to 5.5 min the gradient was held at 10% Solvent A. Hereafter, the column was re‐equilibrated to initial conditions for an additional 2.5 min. The flow was 0.6 mL/min. All analytes were detected in positive ion multiple reaction monitoring (MRM) mode. The following quantifier ion‐transitions were monitored: tryptophan (m/z = 205 → 118), kynurenine (m/z = 209 → 192), kynurenic acid (m/z = 190 → 144), 3OH kynurenine (m/z = 225 → 208), anthranilic acid (m/z = 138 → 120), picolinic acid (m/z = 124 → 78), quinolinic acid (m/z = 168 → 78), 3‐OH anthranilic acid (m/z = 154 → 80), and serotonin (m/z = 177 → 115). The following ion transitions were used for the isotope labeled internal standards: d5‐tryptophan (m/z = 210 → 147), 13C3,15N‐3OH kynurenine (m/z = 229 → 110), d5‐kynurenic acid (m/z = 195 → 149), 13C4,15N‐quinolinic acid (m/z = 173 → 81), 13C6‐kynurenine (m/z = 215 → 152), 13C6‐anthranilic acid (m/z = 144 → 98), d4‐picolinic acid (m/z = 128 → 82), and d4‐serotonin (m/z = 181 → 164).

### 2.3 Statistical analysis

Metabolite concentrations were log10‐transformed and batch‐adjusted separately for each time point using the standardization process of the R package *batchma*. Subjects missing a measurement for one timepoint, but with measures for the remaining timepoints, had the value imputed using the R package *missForest*. Kruskal–Wallis rank sum tests confirmed that there were not significant differences between any metabolites that showed a missing value for the observed and imputed data. Inspection of the metabolite distributions suggested a preference for nonparametric tests.

For the case–control analysis, we used linear models of log10‐transformed metabolites as outcome and case status as predictor to test for differences of baseline metabolites between cases and controls. We considered the covariates of sex, age, and weight and performed a forward/backward selection process to identify models with minimum Bayesian information criterion (BIC).

For the time series, a Friedman test was used as a nonparametric alternative to repeated measures analysis of variance (ANOVA). Any metabolites that showed a significant result in the Friedman test were followed‐up with pairwise WRS tests, adjusting for multiple testing using a false discovery rate (FDR) <0.05 to indicate significance.

Metabolites at baseline (pre‐S2P) were tested for relationships with cardiac catheterization measures of preoperative physiology (mPAP, PVR, SaO_2_) using linear models. Model selection proceeded via a forward/backward selection process that minimized the BIC from an initial set of covariates of sex, age on day of surgery, weight, and ventricular morphology.

Metabolites at each timepoint were tested for relationships with postoperative outcomes (hospital LOS, hypoxemia burden, and intubation duration). Hospital LOS was modeled using a zero‐truncated, negative binomial model due to the count nature of the data, the presence of over‐dispersion, and the absence of any subjects who stayed 0 days (min: 4 days). Model diagnostics confirmed this approach to be appropriate. Hypoxemia burden and intubation duration were log‐transformed to approximate normality and reduce influence of outliers then modeled using a linear model. For each postoperative outcome, model selection proceeded by a stepwise selection process to search for the model that minimized BIC for linear models and Akaike information criterion (AIC) for the zero‐truncated, negative binomial models. Models were selected from the initial covariates of age, weight, sex, ventricular morphology, and pre‐op physiology (mPAP, PVR, and SaO_2_). The reduced model for each postoperative outcome included any covariates that were identified in these final models for the metabolites.

## 3 RESULTS

A total of 75 infants with SVHD and 41 biventricular controls were enrolled between March 2018 and October 2021. Group demographics and baseline characteristics are presented in Table 1. The control group, on average, was older and had a greater weight. For the SVHD group, pre‐S2P cardiac catheterization data demonstrated median mPAP of 13 mmHg, PVR of 2.0 Woods units * meter squared, and SaO_2_ of 74%. Post‐S2P, the median hospital LOS was 7 days, hypoxemia burden was 4.3% in the first 48 h, and duration of intubation was 16 h.

**TABLE 1:** Demographics and baseline characteristics.

	Total (*N* = 113)	SVHD (*N* = 72)	Control (*N* = 41)	*p* Value
Male sex	68 (60%)	42 (58%)	26 (63%)	0.6
Age (months)	5.1 (4.4–6.7)	4.6 (2.2–5.2)	6.9 (5.9–10.0)	**<0.001**
Weight (kilograms)	6.3 (5.6–7.6)	5.7 (5.4–6.3)	7.7 (7.1–8.6)	**<0.001**
Cardiac morphology				
Dominant right ventricle		55 (76%)		
Dominant left ventricle		17 (24%)		
Prior interventions				
Norwood + shunt		44 (61%)		
Shunt only		10 (14%)		
Main pulmonary artery band		6 (8%)		
Other		9 (13%)		
Preoperative clinical data				
Mean PA pressure (mm Hg)		13 (12–15)		
Pulmonary vascular resistance (iWU)		2.0 (1.6–2.6)		
Arterial oxygen saturation (%)		74 (70–77)		
Postoperative clinical data				
Hospital length of stay (days)		7 (5–19)		
Hypoxemia burden (%)		4.3 (2.4–11.6)		
Duration of intubation (hours)		16 (8–32)		

*Note*: Data are presented as *N* (%) or median (interquartile range). Significant *p*‐values are shown in bold.

Abbreviations: iWU, indexed Wood's units; mm Hg, millimeters of mercury; PA, pulmonary arterial; SVHD, single ventricle heart disease.

### 3.1 Interstage metabolite concentrations versus controls

**FIGURE 2:**
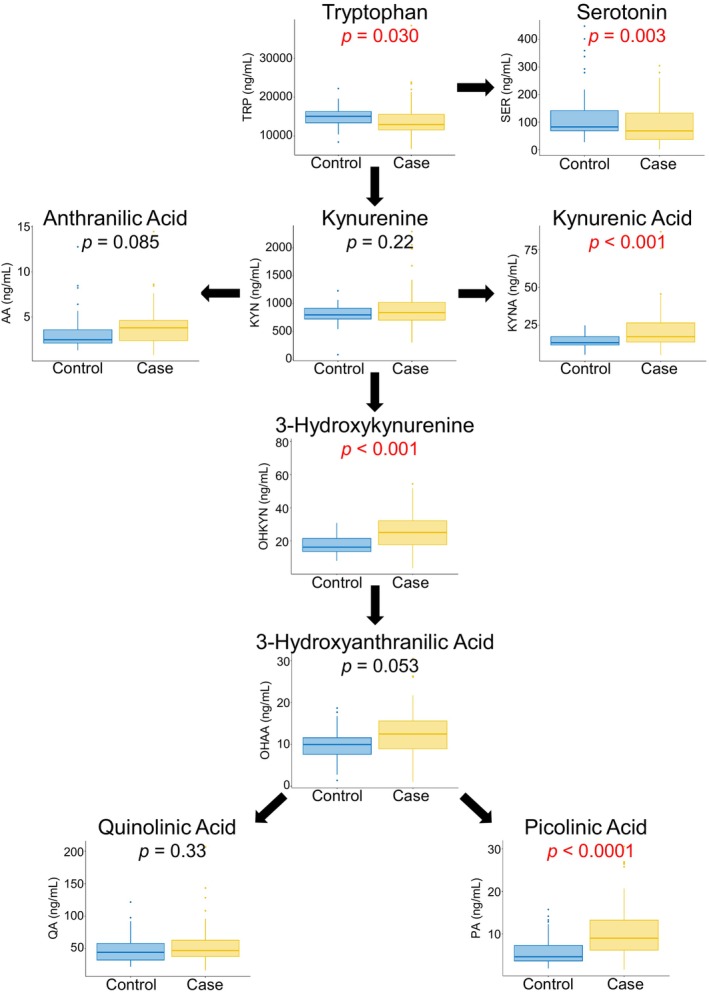
Case–Control Comparison. Linear models of log10‐transformed metabolites as outcome and case status as predictor were used to test for differences in baseline metabolite levels between SVHD cases (*n* = 72) and controls (*n* = 41). The boxes depict the median (bolded line) and the interquartile range.

Metabolite concentrations differed between SVHD subjects and controls for 5 of the 9 KP metabolites (Figure 2). SVHD infants demonstrated lower concentrations of tryptophan and serotonin, and higher concentrations of kynurenic acid, 3‐hydroxykynurenine, and picolinic acid. Concentrations of kynurenine, anthranilic acid, 3‐hydroxyanthranilic acid, and quinolinic acid did not differ by group. Interaction terms including the individual metabolites with each of sex, age, and weight were not significantly associated with case–control status, suggesting the relationship between case–control status and the metabolites do not significantly differ by the variables tested. The only exception was picolinic acid, which varied by sex: for cases picolinic acid levels were higher among females than males (*p* = 0.017) while for controls picolinic acid levels were higher among males (*p* = 0.046). Addition of sex, age, or weight did not improve the model fit for any of the metabolites except PA, where the observed sex differences are noted above, and so we present unadjusted results in our primary analysis. In the Supplemental Data, we include results of the linear regression models including sex (Table S1), weight (Table S2), and age (Table S3) as covariates.

### 3.2 Interstage metabolite relationships with pre‐S2P physiology

SVHD interstage KP metabolite concentrations were compared to pre‐S2P cardiac‐catheterization‐derived physiology. Lower anthranilic acid concentration was associated with higher pre‐S2P mPAP (10‐fold decrease in anthranilic acid concentration was associated with a 1.8 mmHg increase in mPAP, *p* = 0.014) and higher PVR (10‐fold decrease in anthranilic acid concentration was associated with a 0.6 iWU increase in PVR, *p* = 0.011). Higher 3‐hydroxyanthranilic acid and kynurenic acid concentrations were associated with lower SaO_2_ (10‐fold increase in 3‐hydroxyanthranilic acid concentration was associated with a 2.5% decrease in SaO_2_, *p* = 0.008; 10‐fold increase in kynurenic acid concentration was associated with a 2.4% decrease in SaO2, *p* = 0.019). No other interstage SVHD KP metabolite concentrations were associated with pre‐S2P physiology.

### 3.3 Postoperative changes in SVHD metabolite concentrations following S2P

**FIGURE 3:**
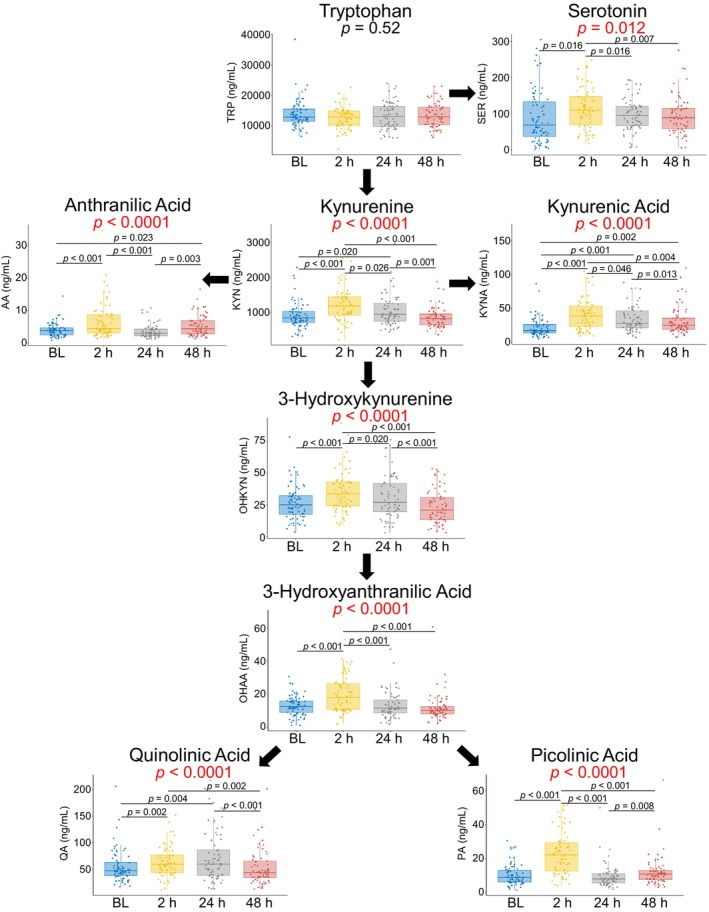
Time Series in SVHD Cases. *p* values shown in red indicate significance for Friedman Tests to assess for differences in metabolite concentrations across the timepoints (nonparametric equivalent of a repeated‐measures ANOVA). Significant results on follow‐up pairwise Wilcoxon‐Rank Sum tests, adjusted for multiple comparisons with FDR, are shown by the significance bars and black *p* values. The boxes depict the median (bolded line) and the interquartile range. BL, baseline.

All KP metabolites, except tryptophan, demonstrated postoperative concentration changes compared to baseline (prior to S2P) (Figure 3). Throughout the pathway, metabolite concentrations were most different from baseline at 2 h post‐S2P, and then slowly returned toward baseline. Serotonin, anthranilic acid, 3‐hydroxykynurenine, 3‐hydroxyanthranilic acid, and picolinic acid returned to baseline by 24 h. Kynurenine and quinolinic acid returned to baseline by 48 h. Kynurenic acid was the only metabolite that had not returned to baseline at 48 h post‐S2P. The metabolites arranged from the largest to smallest percent change at their peak are as follows: kynurenic acid (65% increase), picolinic acid (64% increase), 3‐hydroxyanthranilic acid (57% increase), anthranilic acid (47% increase), serotonin (41% increase), 3‐hydroxykynurenine (25% increase), kynurenine (21% increase), and quinolinic acid (16% increase).

### 3.4 Metabolite concentration relationships with post‐S2P clinical outcomes

The first outcome we examined was postoperative hospital LOS (Table 2). After stepwise multivariate regression model selection (tested covariates listed in methods section), the final model controlled for age, weight, and ventricular morphology. Among baseline (pre‐S2P) metabolites, increased picolinic acid was associated with longer postoperative LOS. Post‐S2P, decreased serotonin at 2, 24, and 48 h and increased kynurenic acid at 24 and 48 h were associated with longer postoperative LOS.

**TABLE 2:** Hospital length of stay.

	Baseline	2 h	24 h	48 h
Tryptophan	0.41 [0.39–4.38] *p* = 0.463	2.28 [0.36–14.61] *p* = 0.383	0.38 [0.07–2.14] *p* = 0.274	0.12 [0.01–1.02] *p* = 0.052
Serotonin	0.60 [0.34–1.07] *p* = 0.084	**0.19 [0.07–0.49]** ***p* = 0.001**	**0.31 [0.14–0.71]** ***p* = 0.006**	**0.15 [0.06–0.37]** ***p* < 0.001**
Kynurenine	0.55 [0.10–3.12] *p* = 0.498	1.85 [0.42–8.21] *p* = 0.417	1.62 [0.33–7.99] *p* = 0.555	0.89 [0.12–6.44] *p* = 0.905
Kynurenic acid	0.85 [0.24–2.99] *p* = 0.805	1.87 [0.73–4.75] *p* = 0.190	**3.30 [1.09–10.00]** ***p* = 0.035**	**4.18 [1.15–15.11]** ***p* = 0.029**
Anthranilic acid	1.95 [0.53–7.20] *p* = 0.316	1.80 [0.75–4.29] *p* = 0.187	2.15 [0.66–7.05] *p* = 0.206	1.06 [0.36–3.17] *p* = 0.913
3‐Hydroxykynurenine	0.74 [0.22–2.48] *p* = 0.626	0.53 [0.14–2.03] *p* = 0.352	0.89 [0.32–2.53] *p* = 0.833	0.99 [0.33–2.96] *p* = 0.993
3‐Hydroxyanthranilic acid	0.75 [0.26–2.15] *p* = 0.596	1.67 [0.61–4.56] p = 0.316	1.68 [0.62–4.55] *p* = 0.306	2.00 [0.61–6.60] *p* = 0.253
Quinolinic acid	1.50 [0.36–6.25] *p* = 0.578	2.01 [0.59–6.89] *p* = 0.266	1.91 [0.68–5.31] *p* = 0.217	1.54 [0.46–5.21] *p* = 0.484
Picolinic acid	**3.61 [1.01–12.90]** ***p* = 0.048**	1.53 [0.54–4.35] *p* = 0.429	1.70 [0.57–5.08] *p* = 0.340	1.06 [0.31–3.65] *p* = 0.926

*Note*: Data are presented as incident rate ratio (IRR) with [confidence interval]. A 10‐fold increase in metabolite concentration is associated with IRR times the hospital length of stay (e.g., a 10‐fold increase in kynurenic acid concentration at 24 h is associated with a 3.30‐fold increase in hospital length of stay). Thus, IRR >1 indicates an increase in hospital length of stay and IRR <1 indicates a reduction in hospital length of stay given an increase in metabolite concentration. Significant associations are shown in bold.

Next, we examined hypoxemia burden in the first 48 postoperative hours (Table 3). After stepwise multivariate regression model selection (tested covariates listed in methods section), the final model controlled for baseline oxygen saturation. Increased kynurenic acid at 24 and 48 h and increased 3‐hydroxykynurenine at 24 h were associated with greater hypoxemia burden. There were no significant relationships between hypoxemia burden and metabolite concentrations at baseline and 2 h post‐S2P.

**TABLE 3:** Hypoxemia burden.

	Baseline	2 h	24 h	48 h
Tryptophan	0.76 [0.05–12.71] *p* = 0.849	0.44 [0.04–4.71] *p* = 0.491	0.13 [0.01–1.32] *p* = 0.082	0.14 [0.01–2.02] *p* = 0.144
Serotonin	0.70 [0.28–1.76] *p* = 0.437	1.56 [0.41–5.90] *p* = 0.510	0.98 [0.30–3.27] *p* = 0.977	0.75 [0.23–2.49] *p* = 0.638
Kynurenine	2.40 [0.28–20.36] *p* = 0.417	1.11 [0.17–7.24] *p* = 0.910	1.46 [0.21–10.15] *p* = 0.700	0.64 [0.06–6.63] *p* = 0.706
Kynurenic acid	0.66 [0.15–3.00] *p* = 0.589	1.92 [0.57–6.41] *p* = 0.283	**6.48 [1.68–25.01]** ***p* = 0.008**	**4.86 [1.18–19.94]** ***p* = 0.029**
Anthranilic acid	0.49 [0.10–2.56] *p* = 0.394	0.89 [0.29–2.75] *p* = 0.831	1.35 [0.01–6.03] *p* = 0.690	0.36 [0.09–1.50] *p* = 0.156
3‐Hydroxykynurenine	1.60 [0.34–7.49] *p* = 0.544	1.26 [0.20–7.77] *p* = 0.803	**3.80 [1.03–14.05]** ***p* = 0.046**	2.96 [0.84–10.43] *p* = 0.090
3‐Hydroxyanthranilic acid	0.69 [0.17–2.88] *p* = 0.608	1.70 [0.47–6.21] *p* = 0.413	2.80 [0.73–10.80] *p* = 0.132	1.26 [0.29–5.46] *p* = 0.756
Quinolinic acid	2.46 [0.40–14.97] *p* = 0.322	1.16 [0.22–6.14] *p* = 0.857	3.44 [0.97–12.21] *p* = 0.056	2.91 [0.65–12.97] *p* = 0.157
Picolinic acid	2.18 [0.48–9.89] *p* = 0.306	0.93 [0.22–3.81] *p* = 0.913	1.17 [0.29–4.67] *p* = 0.821	0.72 [0.15–3.46] *p* = 0.674

*Note*: Data are presented as a coefficient with [confidence interval]. A 10‐fold increase in metabolite concentration is associated with coefficient times the hypoxemia burden (e.g., a 10‐fold increase in kynurenic acid concentration at 24 h is associated with a 6.48‐fold increase in hypoxemia burden). Thus, a coefficient >1 indicates an increase in hypoxemia burden and a coefficient <1 indicates a reduction in hypoxemia burden given an increase in metabolite concentration. Significant associations are shown in bold.

Finally, we examined duration of intubation post‐S2P (Table 4). After stepwise multivariate regression model selection (tested covariates listed in methods section), the final model controlled for age. Among baseline (pre‐S2P) metabolites, increased kynurenine and decreased anthranilic acid were associated with a longer intubation duration. Post‐S2P, decreased serotonin at 2 h, increased kynurenic acid at 24 and 48 h, decreased tryptophan and increased quinolinic acid at 24 h, and decreased anthranilic acid and increased 3‐hydroxykynurenine at 48 h were all associated with longer intubation duration.

**TABLE 4:** Intubation duration.

	Baseline	2 h	24 h	48 h
Tryptophan	8.29 [0.13–530.65] *p* = 0.313	5.76 [0.20–162.33] *p* = 0.298	**0.02 [<0.01–0.26]** ***p* = 0.002**	0.03 [<0.01–1.26] *p* = 0.065
Serotonin	0.60 [0.20–1.75] *p* = 0.343	**0.11 [0.02–0.62]** ***p* = 0.013**	0.56 [0.17–1.91] *p* = 0.351	0.39 [0.07–2.14] *p* = 0.272
Kynurenine	**40.38 [2.02–805.99]** ***p* = 0.016**	4.46 [0.32–62.97] *p* = 0.263	2.05 [0.24–17.46] *p* = 0.670	17.39 [0.65–468.53] *p* = 0.088
Kynurenic acid	1.25 [0.14–11.16] *p* = 0.840	2.89 [0.53–15.68] *p* = 0.214	**7.07 [1.48–33.90]** ***p* = 0.015**	**21.81 [2.88–164.93]** ***p* = 0.003**
Anthranilic acid	**0.09 [0.01–0.82]** ***p* = 0.033**	3.20 [0.67–15.35] *p* = 0.143	2.52 [0.46–13.94] *p* = 0.284	**0.08 [0.01–0.47]** **p = 0.006**
3‐Hydroxykynurenine	0.72 [0.08–6.32] *p* = 0.764	2.98 [0.27–32.89] *p* = 0.366	2.63 [0.64–10.77] *p* = 0.177	**6.88 [1.20–39.38]** ***p* = 0.031**
3‐Hydroxyanthranilic acid	2.42 [0.37–15.92] p = 0.351	2.06 [0.34–12.49] *p* = 0.427	3.06 [0.74–12.65] *p* = 0.120	4.29 [0.52–35.70] *p* = 0.174
Quinolinic acid	1.34 [0.10–18.14] *p* = 0.825	4.53 [0.49–41.47] *p* = 0.178	**6.12 [1.58–23.71]** ***p* = 0.009**	2.63 [0.33–21.17] *p* = 0.357
Picolinic acid	0.99 [0.11–8.75] *p* = 0.994	2.98 [0.46–19.25] *p* = 0.248	1.21 [0.25–5.96] *p* = 0.811	0.80 [0.09–7.20] *p* = 0.836

*Note*: Data are presented as a coefficient with [confidence interval]. A 10‐fold increase in metabolite concentration is associated with coefficient times the intubation duration (e.g., a 10‐fold increase in kynurenic acid concentration at 24 h is associated with a 7.07‐fold increase in intubation duration). Thus, a coefficient >1 indicates an increase in intubation duration and a coefficient <1 indicates a reduction in intubation duration given an increase in metabolite concentration. Significant associations are shown in bold.

## 4 DISCUSSION

Infants with SVHD have variable outcomes following S2P, with some experiencing significant hypoxemia in the immediate postoperative period. Our study sought to delineate the association of tryptophan catabolism with both preoperative physiology and postoperative outcomes surrounding S2P. We performed quantitative, perioperative mapping of the KP, demonstrating abnormal tryptophan catabolism in interstage infants and characterizing KP metabolite concentration trajectories in response to CPB during S2P. We found a small number of associations between interstage KP metabolite concentrations and pre‐S2P physiology. Interstage metabolite concentrations were poorly correlated with post‐S2P outcomes. Following S2P, peak metabolite concentrations (at 2 h) also poorly correlated with outcomes, suggesting that the magnitude of the KP response to CPB did not associate with markers of adjusting to S2P physiology. The exception to this was serotonin. A lower serotonin concentration at 2 h (i.e., a blunted peak) was associated with worse outcomes for two of our criteria: hospital LOS and intubation duration. At 24 and 48 h post‐S2P, there were multiple metabolites that correlated with outcomes. Most notably, elevated kynurenic acid levels at both 24 and 48 h were associated with worse results for all three outcomes tested. Additionally, kynurenic acid was the only metabolite that did not return to baseline by 48 h. This suggests that a delay in the return to baseline after the metabolic peak may be most predictive of poor adjustment to S2P physiology.

### 4.1 Interstage KP activation

This is the first study to quantitatively map the KP of tryptophan catabolism in interstage SVHD infants. The interstage period is a time of particular vulnerability for children with SVHD, corresponding to the highest risk time period for mortality (Kaplinski et al., 2020). The current study demonstrates that exposure to interstage physiology is coincident with baseline KP differences between interstage infants and healthy controls including reduced tryptophan substrate, reduced serotonin, and increased KP metabolites. These results indicate increased KP activity compared to control infants. KP metabolites have wide‐ranging activities and may have implications for interstage morbidity not specifically measured in this study. KP activity is highly correlated with inflammation and leads to immune dysregulation (Wirthgen et al., 2018). Further, the KP is known to include both neurotoxic and neuroprotectant metabolites (Savitz, 2020).

The significant association between multiple KP metabolites and pre‐S2P hemodynamic parameters further highlights the potential importance of the KP to interstage SVHD physiology. Decreased anthranilic acid levels were associated with increased mPAP and PVR prior to S2P. Anthranilic acid function in humans is not well studied and mostly limited to observational correlation with neurologic disorders such as schizophrenia, stroke, migraines, cluster headaches, and depression (Darlington et al., 2010; Oxenkrug et al., 2016; Shaw et al., 2023). Given its correlation with elevated mPAP and PVR in our study, this may be an important area of future research for the SVHD population. In addition to the associations with anthranilic acid, elevated kynurenic acid and 3‐hydroxyanthranilic acid were both associated with lower SaO_2_. Elevated kynurenic acid was previously shown to correlate with a greater hypoxemia burden in premature infants without heart disease (MacFarlane et al., 2023), although the mechanism is unknown. Interestingly, 3‐hydroxyanthranilic acid is known to inhibit synthesis of endogenous nitric oxide, a key pulmonary vasodilator (Oh et al., 2004). The mechanistic link between KP alterations and interstage physiology is an important area for further investigation.

### 4.2 KP response to cardiopulmonary bypass

The results of our study overwhelmingly support increased tryptophan catabolism activity in the immediate postoperative period as all metabolites (except tryptophan itself) demonstrated a peak at 2 h with a subsequent return to baseline. Of note, although we refer to the peak at 2 h, we are limited by the timepoints we measured. In reality, the peak may have occurred at any time from the start of surgery through prior to 24 h post‐op. This is the first study to quantitatively map the KP in children following CPB surgery. Two prior studies in adults undergoing CPB are at odds with each other. Kotlinska‐Hasiec et al. (2015) demonstrated increased kynurenic acid concentration immediately following surgery, consistent with our results, but they did not map the remainder of the pathway. In contrast, Stieger et al. (2024) demonstrated a reduction in the levels of multiple KP metabolites intraoperatively (blood drawn at discontinuation of CPB). Unlike our cohort, theirs had a reduction in tryptophan concentration, indicating a reduction in KP substrate. And thus, the nutritional and baseline metabolic state prior to CPB surgery may have differed between groups, or tryptophan levels could be influenced by different CPB priming techniques (blood prime in pediatrics, clear prime in adults). As we have previously demonstrated, an elevated kynurenic acid level after pediatric CPB surgery is associated with increased mortality and poor outcomes for infants with any cardiac diagnosis (Davidson et al., 2018), and thus quantitative mapping of the KP would be interesting in all populations of pediatric congenital heart disease.

### 4.3 Postoperative circulating kynurenic acid and serotonin

Prolonged elevation of kynurenic acid after S2P identified in our study was associated with poorer outcomes, and the link with greater postoperative hypoxemia burden is most suggestive of coincident elevated PVR. In SVHD patients undergoing S2P, an important mechanism of postoperative hypoxemia is elevated resistance through the pulmonary vascular bed, often due to underdeveloped pulmonary vasculature (Gewillig et al., 2010). On top of this anatomic substrate, CPB is a profound inflammatory stressor on the body which results in microvascular dysfunction system‐wide and a specific propensity for pulmonary vasoconstriction (Kant et al., 2023; Sato et al., 2000). Although our study does not directly measure postoperative pulmonary vascular resistance, the association between delayed kynurenic acid clearance and increased hypoxemia burden could be explained by elevated pulmonary vascular resistance leading to decreased pulmonary blood flow. In mice and adults with pulmonary hypertension, increased KP activity and elevated serum kynurenic acid levels were observed early in the development of pulmonary hypertension (Cai et al., 2022; Simpson, Ambade, et al., 2023), suggesting a potential biologic link. Another study demonstrated increased KP activity in the form of increased NAD+ concentrations in the lungs of rodents with acute lung injury causing pulmonary hypertension (Cai et al., 2022). Elevated NAD+ is believed to be beneficial in compensating for pulmonary hypertension, providing modulation of both mitochondrial dysfunction and inflammation. Further investigation is required to determine whether KP activity is a beneficial compensatory mechanism in the setting of pulmonary hypertension, or whether elevated kynurenic acid levels may contribute to pathophysiology and poor outcomes.

Lower serotonin concentrations at 2 h post‐S2P were also associated with worse outcomes for two of our three outcome measures (hospital LOS and intubation duration). As outlined in Figure 1, serotonin has numerous effects on physiology, affecting nearly every organ system in the body. Most interesting for our SVHD population are the effects on pulmonary vascular resistance. The lungs are an important site of serotonin synthesis, uptake, and metabolism. Broadly, increased serotonin uptake in the lungs leads to vasoconstriction and smooth muscle proliferation underlying pulmonary hypertension (Archambault & Delaney, 2023). In pigs, the pulmonary microvasculature demonstrates vasoconstriction when exposed to serotonin after CPB (Sato et al., 2000). We observed lower circulating serotonin levels in patients with worse outcomes, which could potentially represent greater uptake in the lungs, leading to higher PVR and worse outcomes. Alternatively, given the lungs' role in serotonin synthesis, if patients who were struggling post‐bypass had overall less pulmonary blood flow, we may see a lower circulating peak due to tissue accumulation with reduced perfusion or a compensatory decrease in serotonin production. A blunted serotonin peak may also represent decreased catabolism of tryptophan through the serotonin pathway as increased flux through the KP reroutes tryptophan away from serotonin. Because the KP is activated by inflammation, this rerouting may mirror the intensity of the systemic inflammatory response to CPB, thus those with a greater inflammatory response would manifest lower serotonin levels at the metabolic peak.

### 4.4 Limitations

Limitations of the current study include those inherent to a single‐center study, mainly that outcomes were influenced by specific practices at our institution and generalizability to other centers was not assessed. It is also important to note that our center is at moderately increased altitude (5403 ft) and this may reduce the generalizability of our findings. By design, our study measured associations, not causation. We believe our data raise interesting questions for subsequent mechanistic research.

Limitations also exist on a more granular level. Serum samples were analyzed in multiple batches for pathway mapping. This required statistical standardization across batches, which we then confirmed for accuracy by multiple methods. Visual assessments with boxplots, quantile‐quantile plots, principal component analysis plots, and tests of global explained variance with partial redundancy analysis, scaled R (Blinder et al., 2012), and ANOVA all confirmed the adequacy of our batch correction. Another limitation was the difference in size and age between cases and controls. To address this, we assessed age and weight as covariates in our case–control analysis. Both variables had insignificant interaction terms, there were minimal interpretation differences between the models, and the best model fit (lowest BIC) was observed for the univariate (unadjusted) models. Thus, we do not believe that the age and weight differences between groups significantly impacted the case–control comparison in this study and we chose to present the univariate model (that did not include age or weight) as the primary analysis.

## 5 CONCLUSIONS

KP activity in SVHD infants is abnormal in the interstage period. Further, the KP response to CPB and S2P surgery reveals multiple associations between post‐S2P KP metabolite levels and clinical outcomes. Most strikingly, increased kynurenic acid at 24 and 48 h, as well as reduced serotonin at 2 h, are associated with multiple measures of poor outcomes. We believe the data presented in this manuscript to be an important step prior to future mechanistic studies into the metabolic importance of KP activation in the vulnerable SVHD population.

## AUTHOR CONTRIBUTIONS

BSF and JAD conceived and designed research; BSF, LK, and TL performed experiments; SN, CM, JR, BSF, and JAD analyzed data; SN, CM, JR, JK, BSF, and JAD interpreted results of experiments; SN and JR prepared figures; JR, SN, BSF, and JAD drafted manuscript; all authors edited and revised manuscript; all authors approved final version of manuscript.

## FUNDING INFORMATION

This study was supported by the American Heart Association—AHA20CDA35310498 (Frank), AHA23CDA1038247 (Romanowicz), and AHA18IPA34170070 (Davidson); the National Institutes of Health—NIH/NCATS Colorado CTSA, No. UL1 TR001082 and UL1 TR002535, NIH/NHLBI K23HL123634 (Frank), and R01HL156936 (Davidson); and the Jayden DeLuca Foundation. There was no relationship with industry associated with this manuscript.

## CONFLICT OF INTEREST STATEMENT

The authors declare no conflicts of interest.

## ETHICS STATEMENT

Permission to conduct the study was granted by the Colorado Multiple Institutional Review Board. Written informed consent was obtained from each subject's legal guardian at enrollment for both cases and controls. The research was conducted in accordance with the principles embodied in the Declaration of Helsinki and in accordance with local statutory requirements.

## Supporting information


Tables S1–S3.

## Data Availability

The data that support the findings of this study are available from the corresponding author, JR, upon reasonable request.
