# Supplementary material for: Perturbations of tryptophan catabolism via the kynurenine pathway are associated with stage 2 postoperative outcomes in single ventricle heart disease
Source: Physiol Rep. 2024 Nov 24;12(22):e70133. doi: 10.14814/phy2.70133 (PMC11586103; doi:10.14814/phy2.70133)
Supplement: Supplementary file 1 — Tables S1–S3. [file PHY2-12-e70133-s001.docx]

Supplemental Table 1. Cases vs Controls Linear Regression Model with Sex as a Covariate.

|  | Estimate | Multiple Change Estimate | Multiple Change Estimate CI | p-value | FDR adjusted p-value |
| --- | --- | --- | --- | --- | --- |
| Tryptophan | -0.049 | 0.893 | 0.810-0.985 | **0.023** | **0.042** |
| Kynurenine | 0.039 | 1.094 | 0.941-1.273 | 0.240 | 0.270 |
| Kynurenic Acid | 0.137 | 1.371 | 1.146-1.640 | **<0.001** | **0.0021** |
| 3-Hydroxykynurenine | 0.161 | 1.449 | 1.199-1.750 | **<0.001** | **<0.001** |
| Anthranilic Acid | 0.074 | 1.187 | 0.975-1.445 | 0.087 | 0.112 |
| Picolinic Acid | 0.242 | 1.744 | 1.399-2.174 | **<0.0001** | **<0.0001** |
| Quinolinic Acid | 0.039 | 1.094 | 0.918-1.303 | 0.314 | 0.314 |
| 3-Hydroxyanthranilic Acid | 0.091 | 1.232 | 0.989-1.533 | 0.062 | 0.093 |
| Serotonin | -0.240 | 0.575 | 0.398-0.833 | **0.0037** | **0.0084** |

A change from control to case is associated with an “Estimate” unit change in log10(metabolite). Cases have metabolite concentrations that are “Multiple Change Estimate” times higher than controls. CI = 95% confidence interval. FDR = false discovery rate.

Supplemental Table 2. Cases vs Controls Linear Regression Model with Weight as a Covariate.

|  | Estimate | Multiple Change Estimate | Multiple Change Estimate CI | p-value | FDR adjusted p-value |
| --- | --- | --- | --- | --- | --- |
| Tryptophan | -0.076 | 0.839 | 0.740-0.952 | **0.0067** | **0.015** |
| Kynurenine | 0.045 | 1.109 | 0.912-1.348 | 0.299 | 0.384 |
| Kynurenic Acid | 0.130 | 1.349 | 1.070-1.699 | **0.012** | **0.021** |
| 3-Hydroxykynurenine | 0.148 | 1.406 | 1.103-1.792 | **0.0063** | **0.015** |
| Anthranilic Acid | 0.030 | 1.072 | 0.834-1.377 | 0.584 | 0.657 |
| Picolinic Acid | 0.191 | 1.553 | 1.172-2.059 | **0.0025** | **0.011** |
| Quinolinic Acid | -0.002 | 0.995 | 0.796-1.245 | 0.966 | 0.966 |
| 3-Hydroxyanthranilic Acid | 0.124 | 1.332 | 1.003-1.769 | **0.048** | 0.072 |
| Serotonin | -0.329 | 0.469 | 0.293-0.751 | **0.0019** | **0.011** |

A change from control to case is associated with an “Estimate” unit change in log10(metabolite). Cases have metabolite concentrations that are “Multiple Change Estimate” times higher than controls. CI = 95% confidence interval. FDR = false discovery rate.

Supplemental Table 3. Cases vs Controls Linear Regression Model with Age as a Covariate.

|  | Estimate | Multiple Change Estimate | Multiple Change Estimate CI | p-value | FDR adjusted p-value |
| --- | --- | --- | --- | --- | --- |
| Tryptophan | -0.037 | 0.919 | 0.818-1.033 | 0.156 | 0.201 |
| Kynurenine | 0.040 | 1.097 | 0.912-1.320 | 0.324 | 0.365 |
| Kynurenic Acid | 0.119 | 1.317 | 1.058-1.638 | **0.014** | **0.025** |
| 3-Hydroxykynurenine | 0.134 | 1.362 | 1.089-1.705 | **0.0073** | **0.022** |
| Anthranilic Acid | 0.076 | 1.191 | 0.938-1.512 | 0.149 | 0.201 |
| Picolinic Acid | 0.193 | 1.561 | 1.214-2.007 | **<0.001** | **0.0057** |
| Quinolinic Acid | -0.003 | 0.994 | 0.803-1.230 | 0.954 | 0.954 |
| 3-Hydroxyanthranilic Acid | 0.146 | 1.399 | 1.072-1.826 | **0.014** | **0.025** |
| Serotonin | -0.325 | 0.473 | 0.302-0.741 | **0.0013** | **0.0057** |

A change from control to case is associated with an “Estimate” unit change in log10(metabolite). Cases have metabolite concentrations that are “Multiple Change Estimate” times higher than controls. CI = 95% confidence interval. FDR = false discovery rate.
